# Supplementary material for: Persons tested for SAR-CoV-2 at a military treatment facility in Hawaii
Source: PLoS One. 2022 Feb 4;17(2):e0263472. doi: 10.1371/journal.pone.0263472 (PMC8815911; doi:10.1371/journal.pone.0263472)
Supplement: Attachment — Submitted filename: Cover letter.docx [file pone.0263472.s005.docx]

Department of the Army

HEADQUARTERS, TRIPLER ARMY MEDICAL CENTER

1 JARRETT WHITE ROAD

TRIPLER AMC, HAWAII 96859-5000

Dec 30th, 2021

**REPLY TO**

**ATTENTION OF**

Dear Editors,

Thank you for inviting us to submit a revised draft of our manuscript entitled, " Persons tested for SAR-CoV-2 at a military treatment facility in Hawaii" to PLOS ONE. We also appreciate the time and effort you and each of the reviewers have dedicated to providing insightful feedback on ways to strengthen our paper. Thus, it is with great pleasure that we resubmit our article for further consideration. We have incorporated changes that reflect the detailed suggestions you have graciously provided. We also hope that our edits and the responses we provide below satisfactorily address all the issues and concerns you and the reviewers have noted.

To facilitate your review of our revisions, the following is a point-by-point response to the questions and comments delivered in your letter dated Dec 9th, 2021.

EDITOR SUGGESTIONS:

1. Please ensure that your manuscript meets PLOS ONE's style requirements
   - RESPONSE: The editing changes were made based on PLOS ONE’s style requirements.
2. Please ensure that you refer to Figure 7 in your text as, if accepted, production will need this reference to link the reader to the figure.

- RESPONSE: Thank you for bring this up. Figure 7 has been added to the text.

3. Please include captions for your Supporting Information files at the end of your manuscript, and update any in-text citations to match accordingly. Please see our Supporting Information guidelines for more information: <http://journals.plos.org/plosone/s/supporting-information>.

REVIEWER 1 COMMENTS:

4. Figures quality may be improved with high resolution images (minor).
   - RESPONSE: We increased the image quality of our graphs.
5. The English of manuscript can be refined (minor).
   - RESPONSE: We review the manuscript and refined the English. Grammar changes has been made. Thank you for your feedback.
6. It is required to provide an additional illustrative figure as to highlight the summary or prospect of this study.
   - RESPONSE: Thank you for this suggestion; however, we already provide 7 graphs with our results.
7. More discussion needed to explain the findings.
   - RESPONSE: We included a discussion section.
8. Authors should cite some research to strengthen their hypothesis
   - Response: Thank you for your suggestion, this has been addressed. We included several studies to support our findings.

REVIEWER 2 COMMENTS:

9. The bar graphs in the manuscript (figure1,2,& 3) are regular Microsoft excel graphs. They should be changed for publication.
   - RESPONSE: All figures are Microsoft excel graphs. We are open to suggestions to programs that are free. Meanwhile, we increase the quality of our image to better represents our graphs.
10. Discussion is completely missing from the manuscript. Please discuss our results
    - RESPONSE: Discussion section was added.
11. Figure legends are also missing from the manuscript.
    - RESPONSE: Legends were added
12. Proper reference is not provided in the manuscript.
    - RESPONSE: Reference were added to the manuscript

Again, thank you for giving us the opportunity to strengthen our manuscript with your valuable comments and queries. We have worked hard to incorporate your feedback and hope that these revisions persuade you to accept our submission.

Please address all correspondence concerning this manuscript to me at [Javier.j.barranco@gmail.com](mailto:Javier.j.barranco@gmail.com)

Thank you for your consideration of this manuscript.

Javier Barranco-Trabi, MD

Tripler Army Medical Center

Internal Medicine
